# Identification of Cellular Compositions in Different Microenvironments and Their Potential Impacts on Hematopoietic Stem Cells HSCs Using Single-Cell RNA Sequencing with Systematical Confirmation

**DOI:** 10.3390/life13112157

**Published:** 2023-11-02

**Authors:** Yanan Chi, Guanheng Yang, Chuanliang Guo, Shaoqing Zhang, Lei Hong, Huixiang Tang, Xiao Sang, Jie Wang, Ji Ma, Yan Xue, Fanyi Zeng

**Affiliations:** 1 Department of Histo-Embryology, Genetics and Developmental Biology, Shanghai Jiao Tong University School of Medicine, Shanghai 200025, China; 2 Shanghai Institute of Medical Genetics, Shanghai Children’s Hospital, Shanghai Jiao Tong University School of Medicine, Shanghai 200040, Chinatanghx@shchildren.com.cn (H.T.); sangx2189@shchildren.com.cn (X.S.);; 3 NHC Key Laboratory of Medical Embryogenesis and Developmental Molecular Biology & Shanghai Key Laboratory of Embryo and Reproduction Engineering, Shanghai 200040, China; 4 School of Pharmacy, Macau University of Science and Technology, Macau 999078, China

**Keywords:** hematopoietic stem cells (HSCs), microenvironment, macrophage, fetal liver, single-cell RNA sequencing

## Abstract

Hematopoietic stem cells (HSCs) are stem cells that can differentiate into various blood cells and have long-term self-renewal capacity. At present, HSC transplantation is an effective therapeutic means for many malignant hematological diseases, such as aplastic hematological diseases and autoimmune diseases. The hematopoietic microenvironment affects the proliferation, differentiation, and homeostasis of HSCs. The regulatory effect of the hematopoietic microenvironment on HSCs is complex and has not been thoroughly studied yet. In this study, we focused on mononuclear cells (MNCs), which provided an important microenvironment for HSCs and established a methodological system for identifying cellular composition by means of multiple technologies and methods. First, single-cell RNA sequencing (scRNA-seq) technology was used to investigate the cellular composition of cells originating from different microenvironments during different stages of hematopoiesis, including mouse fetal liver mononuclear cells (FL-MNCs), bone marrow mononuclear cells (BM-MNCs), and *in vitro*-cultured fetal liver stromal cells. Second, bioinformatics analysis showed a higher proportion and stronger proliferation of the HSCs in FL-MNCs than those in BM-MNCs. On the other hand, macrophages in *in vitro*-cultured fetal liver stromal cells were enriched to about 76%. Differential gene expression analysis and Gene Ontology (GO) functional enrichment analysis demonstrated that fetal liver macrophages have strong cell migration and actin skeleton formation capabilities, allowing them to participate in the hematopoietic homeostasis through endocytosis and exocytosis. Last, various validation experiments such as quantitative real-time PCR (qRT-PCR), ELISA, and confocal image assays were performed on randomly selected target genes or proteins secreted by fetal liver macrophages to further demonstrate the potential relationship between HSCs and the cells inhabiting their microenvironment. This system, which integrates multiple methods, could be used to better understand the fate of these specific cells by determining regulation mechanism of both HSCs and macrophages and could also be extended to studies in other cellular models.

## 1. Introduction

Hematopoietic stem cells (HSCs) are pluripotent stem cells that can differentiate into various types of blood cells and have long-term self-renewal capacity [1,2,3]. They are often used clinically to treat hematological diseases such as malignant hematological diseases, aplastic hematological diseases, and autoimmune diseases [4,5,6,7]. The success of hematopoiesis after hematopoietic stem cell transplantation largely depends on the quantity and quality of HSCs infused, and thus obtaining sufficient high-quality HSCs is one of the bottlenecks hindering their clinical application.

It has long been known that the hematopoiesis process in mammals begins with the yolk sac of an early embryo, which produces primitive nucleated red blood cells, megakaryocytes, and macrophages. This primitive hematopoiesis process is limited and cannot produce HSCs [8,9,10] indefinitely. Hematopoiesis then begins in the aorta-gonad-mesonephros (AGM) region and undergoes the process of endothelial-to-hematopoietic transition (EHT), at which point HSCs can induce permanent hematopoiesis [11,12,13,14]; however, the number of HSCs produced is very small. Rapid hematopoiesis occurs during the hematopoietic stage of the fetal liver (FL), where the number of transplantable HSCs in the FL is increased 20 times, rising from 50 to about 1000 from E12.5 to E14.5 [15,16,17]. In the late stage of FL hematopoiesis, the process begins to change from extramedullary to intramedullary hematopoiesis [18,19]. Eventually, hematopoiesis after birth and in adults occurs in the bone marrow (BM) [18,19]. Thus, FL and BM are two crucial developmental sites for HSCs where the dynamic transition of HSCs is coupled to a switch from a proliferative to a predominantly quiescent phenotype. Compared to BM-derived HSCs, FL-derived HSCs have superior engraftment potential and an improved active expansion ability [16]. This prompted us to specifically investigate the difference between FL-derived HSCs and BM-derived HSCs to establish a detailed molecular signature that may clarify the possible underlying mechanism.

Indeed, the generation and maintenance of HSCs require a hematopoietic microenvironment to regulate the proliferation, differentiation, and homeostasis of the HSCs. Therefore, studying the regulatory effect of hematopoietic microenvironment cells on HSCs is of great significance in guiding the research on in vivo hematopoietic reconstruction and the expansion and differentiation induction of HSCs *in vitro*. These heterogeneous cells interact with each other and jointly regulate HSCs. This study of the function of a single cell type may not fully reflect the hematopoietic effect on HSCs. Thus, in this study, 10x Genomic single-cell RNA sequencing (scRNA-seq) was used to sequence cells representing different hematopoietic microenvironments in the FL and BM sites, that are essential for HSCs proliferation and resting, particularly E13.5 fetal liver mononuclear cells (FL-MNCs) and four-week-old mouse bone marrow mononuclear cells (BM-MNCs), respectively. Given the difficulty of obtaining fetal liver samples *in vivo*, mouse fetal liver stromal cells from in vitro culture (passage 3 of *in vitro*-cultured fetal liver sample, FL-P3) were also investigated to explore key factors that affect HSCs functions. The different characteristics of the hematopoietic microenvironment in the FL and BM were examined and analyzed at the cellular and molecular levels to elucidate insights into effective hematopoiesis. This research strategy can also be applied to investigate other cellular models, such as various cancers and their microenvironment cells.

## 2. Materials and Methods

### 2.1. Laboratory Animals

1. C57BL/6 female and male mice were purchased from Shanghai Southern Biological Model Center;
2. Mouse embryos at 13.5d gestation were obtained by breeding C57BL/6 female and male mice.

### 2.2. Experimental Methods

#### 2.2.1. Preparation of FL-MNCs

1. C57BL/6 pregnant mice at 13.5 days of gestation were sacrificed via cervical dislocation. Thirty-three embryos were taken and transferred to the anatomical liquid, and the fetal liver was taken. The added sample diluent was blown repeatedly into cell suspension, and after cell counting, it was slowly added into the supernatant of mononuclear cell separation solution at a ratio of 1:1 and centrifuged for 30 min at 25 °C and 450 g;
2. After centrifugation through a density gradient, all the mononuclear cells in the middle layer were separated, and PBS was added for washing 4 to 5 times. The cells were counted and centrifuged at 1000× *g* for 10 min at 4 °C to obtain fetal liver mononuclear cells. The main materials used in the experiments were centrifuge tubes (Corning, Wujiang, China), dishes (Corning, Wujiang, China), and filters (MiltenyiBiotec, Bergisch Gladbach Cologne, Germany); anatomical liquid (7% FBS in PBS); FBS (Thermo Fisher, Shanghai, China); sample diluent (PBS, Yuanpei, Shanghai, China) and the supernatant of the mononuclear cell separation (Stemcell, Technologies, Vancouver, Canada).

#### 2.2.2. Preparation of BM-MNCs

1. Ten 4-week-old C57BL/6 female mice were sacrificed via cervical dislocation. The femur was taken, and the bone marrow cells were taken into a 1 mL syringe and collected in a 15 mL centrifuge tube. After repeated blowing and homogenization, the mixture was filtered through a 30 μm filter screen into a new 15 mL centrifuge tube. Cells were counted, added at a 1:1 ratio to the supernatant of the mononuclear cell separation, and centrifuged for 30 min at 25 °C and 450 g;
2. After centrifuging through a density gradient, all the mononuclear cells in the middle layer were absorbed, and PBS was added 4–5 times for washing. After cell counting, BM-MNCs were obtained by centrifuging at 4 °C and 1000× *g* for 10 min.

#### 2.2.3. Culture and Collection of FL Stromal Cells

The obtained fetal liver mononuclear cell precipitate was re-suspended with stromal cell culture medium (0.1 mM β-mercaptoethanol, 1% penicillin/streptomycin (*v*/*v*), 15% FBS and high sugar DMEM solution), and inoculated into Petri dishes at a density of 1–2 × 10^6^/cm^2^. After two days, the suspended cells were removed. At up to 95% confluence, the cells were digested into single cells using 0.25% trypsin and collected after three generations of amplification.

#### 2.2.4. Treatment of Cultured Cells

One sample each from the above cell culture preparation was subjected to further treatment to remove dead cells and cell debris using Miltenyi’s dead cell removal kit (Miltenyi Biotec, Bergisch Gladbach Cologne, Germany) after digestion into single cells with 0.25% trypsin at 80–90% confluence. The cell activity was then detected with Trypan blue staining (Thermo Fisher Scientific, Shanghai, China) before scRNA-seq.

#### 2.2.5. 10x Genomics Single-cell Transcription Set Sequencing

The prepared single-cell suspension, 10x Barcode gel beads, and oil drops were added into different channels of Chromium Chip B and placed in the Chromium Controller according to the experimental steps described in the official operating instructions of 10x Genomics Chromium Single Cell 3 Reagent Kitsv3, and then the GEMs (gel beads-in-emulsions) were formed through the microfluidic “double-cross” crossing system. The prepared GEM sample was then subjected to cDNA amplification, and then, through purification and quality inspection, the qualified cDNA sample was enzymatically cut into fragments of about 200–300 bp, and the fragments were subjected to terminal flattening, addition of poly A tail, TSO primer, P5 and P7 linker, and PCR amplification to obtain a DNA library. Finally, high-throughput sequencing of the QC-qualified libraries was performed using the paired-end multiplexing run (150 bp) of the Illumina NovaSeq 6000 sequencing platform (San Diego, CA, USA). The above experimental techniques were provided by LC-Biotechnology Co. Ltd., Hangzhou, China.

#### 2.2.6. Sequencing Data Conversion and Quality Control

Sequencing source files were parsed and converted to raw sequencing data in the FASTQ format using Illumina bcl2fastq software (version 2.20). The raw sequencing data from each sample were then subjected to data filtering, comparison (reference genome: Mus-musculus. GRCm38.p6), and genetic and cellular quantitation using the 10x Genomics official analysis software Cell Ranger (version 3.0.2). The reads for UMIs counting were must be those compared to a single gene, and the number of unique UMI after deduplication is used to represent the gene expression.

#### 2.2.7. Data Processing and Clustering Analysis

The Seurat (version 3.2.3) data analysis R package was used for further quality control of the Cell Ranger processed data. The thresholds of nFeature > 500 and percent.mt < 15 were set for filtering. The gene expression was homogenized using LogNormalize of the NormalizeData function. Then, after the anchor points are found through the SelectIntegrationFeatures function, the three filtered sample data were integrated using the IntegrateData function. Then, principal component analysis (PCA) was used to reduce the dimension, and the first 30 principal components were selected for subsequent clustering and clustering analysis. Then, the cell clusters were identified by the clustering algorithm optimized based on the shared nearest neighbor (SNN) module. Finally, the cells are divided into different subsets using the FindClusters function and visualized using the Uniform Manifold Approximation and Projection (UMAP) method. Raw and processed data analyses were conducted using Lianchuan Nebula. Bioinformatics analysis was performed using the OmicStudio tools “https://www.omicstudio.cn/tool (accessed on 27 May 2019)” [20].

#### 2.2.8. Differentially Expressed Gene Analysis

The FindMarkers function in the R package of Seurat data and the bimodal likelihood ratio statistical test were used to analyze differentially expressed genes between cell subsets or between different sample cell subsets. The screening conditions were set to “min. pct = 0.25” and, and subsequently “p_val_adj < 0.05 & avg_log2FC > 0”. Finally, the expression levels of these differentially expressed genes were analyzed and visualized using the corresponding software.

Differentially expressed genes (exemplified by cytokines) were used to validate the single-cell RNA sequencing analysis result at the protein level using an ELISA assay according to the manufacturer’s instructions. Information on the reagents is as follows: ELISA Kit (ABclonal Technology, Wuhan, China), CCL2 (Cat: RK04218), CCL3 (Cat: RK04218), CCL4 (Cat: RK04212), IL11 (Cat: RK00155), SCF (Cat: RK00389).

#### 2.2.9. Gene Ontology (GO) Enrichment Analysis of Differentially Expressed Genes

The differentially expressed genes in the macrophages cluster (1654 up-regulated genes, see Supplementary Table S6) were screened with the parameter p_Val_Adj < 0.05 and avg_LogFC > 0.25. Then, the clusterProfiler package was used for GO enrichment analysis, with the parameters set as follows: pAdjustMethod = “BH”, pvalueCutoff = 0.05, qvalueCutoff = 0.05. Finally, a bar chart was created presenting the top 10 enrichment results.

#### 2.2.10. Quantitative Real-Time PCR (qRT-PCR)

Total RNA was extracted from MNCs for RT-PCR with RNA-easy Isolation Reagent (Vazyme, Nanjing, China). cDNA libraries for microcellular RNA-seq were also used as templates in the quantitative real-time PCR confirmation assay using HiScript III RT SuperMix (Vazyme, Nanjing, China). qRT-PCR was performed with ChamQ Universal SYBR qPCR Master Mix (Vazyme, Nanjing, China). The sequences of forward and reverse primers applied in qRT-PCR analysis are shown in Supplementary Table S1.

#### 2.2.11. Confocal Image Analysis of Cell Surface Labeling

FL-MNCs were first labeled with F4/80 antibody (BD: Pharmingen, NJ, USA), and then positive cells (F4/80^+^) were isolated using immunomagnetic bead separation method. FL-F4/80^+^ cells were incubated with different primary antibodies and diluted in PBS with 0.2% BSA and 0.1% TritonX-100 and left at 4 °C overnight. The cryosections were incubated with Alexa Fluor secondary antibodies (Invitrogen, CA, USA) for 1 h at RT and then incubated with DAPI for 5 min at RT. After the second round of fixation, cryosections were ready for imaging. Leica DMRXA2 was used to collect immunofluorescence images. The primary antibodies used in our study were as follows: Anti-CD14 antibody (ABclonal technology, Wuhan, China), CD36 (ABclonal technology, Wuhan, China and NRP2 (ABclonal technology, Wuhan, China).

#### 2.2.12. Statistical Analysis

The chi-square test was applied to determine whether there were any significant differences presented in Supplementary Table S2. One-way ANOVA was used to analyze the data in Supplementary Table S1 and the qRT-PCR data. Statistical analysis was performed using SPSS 17.0 software. The graphs were created using GraphPad Prism 9.0.0 software.

## 3. Results

### 3.1. Cellular Composition Analysis

The original sequencing data of samples, including BM-MNCs, FL-MNCs, and *in vitro*-cultured fetal liver stromal cells (FL-P3), were obtained using the Illumina platform followed by quality control analysis using Cell Ranger software data. After quality control, the data were further screened ad filtered using Seurat data analysis package R (version 4.0.3). The statistical results are shown in Supplementary Tables S2–S4.

**Figure 1:**
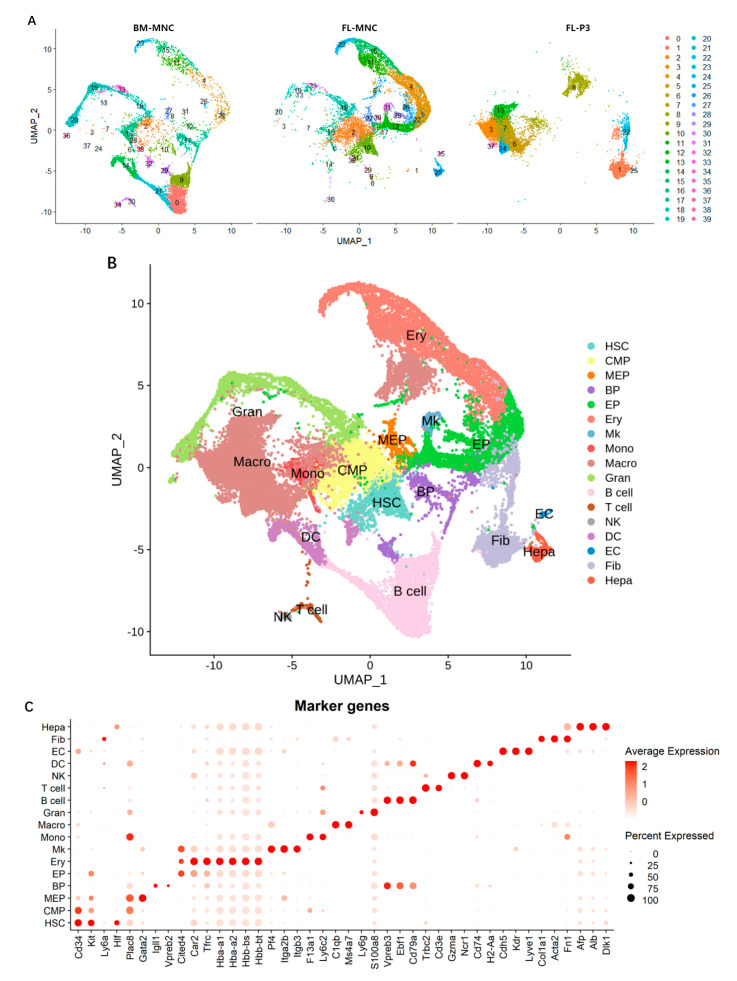
Cell clustering and annotation analysis results of BM-MNC, FL-MNC, and FL-P3 sample integration analysis. (**A**) A UMAP plot showing the results of cell population for the BM-MNC, FL-MNC, and FL-P3 samples, wherein each point represents a cell, and different colors represent different cell subsets. (**B**) UMAP plot showing annotated results for cell subsets, wherein each dot represents a cell, and different colors represents a different cell subsets. (**C**) Bubble chart showing the expression of the marker gene characteristic of various cell subsets, wherein a red color indicates a higher expression level of the gene, and a larger dot indicates a higher expression ratio of the gene in the cell subsets.

Integration analysis was performed on the data sets for the BM-MNC, FL-MNC, and FL-P3 samples. First, the gene expression of the filtered cells was homogenized on a single-cell basis. Then, the cell data of the three samples were integrated and subjected to dimension reduction via PCA. Subsequently, the cells were clustered using the SNN method and grouped under the condition of the parameter “resolution = 1.3”. The results are displayed visually with a UMAP plot. As shown in Figure 1, the cells were separated into 40 cell subsets; the BM-MNC sample contained 36 cell subsets, the FL-MNC sample contained 35 cell subsets, and the FL-P3 sample contained 10 cell subsets (Figure 1A). These 40 cell subsets were used for annotating text. The results for cell subsets and marker gene information are presented in Supplementary Table S5.

A total of 17 cell types were annotated (Figure 1B) and divided into three major categories: (1) 10 types of “myeloid cells”, including hematopoietic Stem cells (HSCs), common myeloid progenitors (CMPs), megakaryocyte and erythrocyte progenitors cells (MEPs), erythroid progenitors (EPs), red blood cells (erythroid, Ery), megakaryocytes (Mk), monocytes (Mono), macrophages (Macro), granulocytes (Gran), and dendritic cells (DCs); (2) 4 types of “lymphocytic cells”, including B lymphocyte progenitors (BPs), B cells, T cells, and natural killer cells (NKs); and (3) 3 types of “other cells”, including endothelial cells (ECs), fibroblast (Fib), and hepatic progenitor cells (hepatoblasts, Hepa). Moreover, the expression status of marker genes in the annotated cell subsets was also noted (Figure 1C).

**Figure 2:**
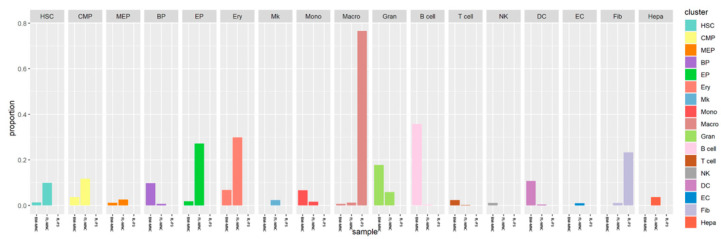
The distribution of each cell cluster in BM-MNC, FL-MNC, and FL-P3 samples.

The proportions of various cell subsets in the BM-MNC, FL-MNC, and FL-P3 samples were analyzed (Figure 2). FL-MNCs appeared to be mainly be composed of 16 out of the 17 cell types, except for NK. This result is similar to the reported composition of human fetal liver cells [21]. Among these cell types, the proportion of hematopoietic stem/progenitor cells (including HSCs, CMP, and MEP) was higher, especially for the HSCs and CMP subsets that reaching 9.95% and 11.75%, respectively. On the other hand, the percentage for the HSCs and CMP subsets in BM-MNCs were 1.34% and 3.63%, indicating a more extensive hematopoiesis process in the FL than in the BM. Further experiments need to be investigated.

The bone marrow hematopoietic microenvironment is comprised of 15 of the 17 cell types, lacking ECs and Hepa. The proportion of lymphocytes was very high, reaching 49.13%, and a particularly higher fraction was seen for the subgroup of B cells reached 35.78%. Additionally, the proportions of Gran, DC, and Mono subsets in the BM-MNCs were also high, accounting for about 10% of the total cells. This could suggest the role of bone marrow in the body’s immune system.

Different from the in vivo environment, the fetal liver stromal cells obtained through in vitro culture were mainly composed of three cell populations: Macro, Fib, and Hepa, accounting for 76.6%, 23.31%, and 0.09% of the total cells, respectively. The highest proportion of macrophages was found in fetal liver stromal cells, whereas the proportion of macrophages in in vivo fetal liver samples was only 1.24%. This may have been due to the efficient amplification of macrophages under our cultural conditions. This in vitro culture might be helpful in studying the three subtypes more in depth in relation to the hematopoietic microenvironment.

### 3.2. Differential Gene Expression Analysis

To further explore the differences between FL-MNC and BM-MNC, the differential expression genes were identified, as shown in Supplementary Table S5, Differential expression analysis of co-existing cell subsets in the FL and BM samples was performed, the results of which are shown in Supplementary Figure S2. Compared with those from the bone marrow, the fetal liver HSCs expressed a higher level of erythroid-cell-specific genes such as *Hbb-bt* and *Hba-a1* (Figure S2A), indicating a stronger erythroid differentiation ability. In addition to HSCs, the CMPs and MEPs in the fetal liver also highly expressed the cell-expansion-related gene *Hmga2* [22,23] and the protein-assembly-related genes *Npm1* [24] and *Ncl* [25] (Figure S2A–C), implying the stronger proliferation capacity in these hematopoietic stem/progenitor cells in fetal liver. This may be related to the rapid proliferation of FL-MNCs and HSCs and their rapid migration to downstream red blood cells and other cells. Notably, the macrophage migration inhibitory factor gene, *Mif*, was highly expressed in these subsets of cells, suggesting that this may ensure the retention of macrophages and play a corresponding role in the surrounding cells.

The interaction between macrophages and HSCs was investigated first by examining the gene expression characteristics of macrophage subsets. A comprehensive analysis of differential expression genes yielded a total of 1654 genes with significantly higher expression of macrophages from FL-MNCs (see Supplementary Table S6). The GO enrichment analysis results for these highly expressed genes regarding three aspects biological process, cell composition, and molecular function are shown in Supplementary Figure S3. Specifically, biological processes refers to enriched processes, including actin filament fabric, bone marrow leukocyte activation, leukocyte migration, regulation of actin cytoskeletal fabric, myeloid cell differentiation and positive regulation of cell adhesion, which are associated with the immune regulation and other functions of macrophages; the cell composition signifies the dissolution of vesicles, lysosomes, endocytosis of vesicles, which are mainly related to the phagocytosis of macrophages; and finally, molecular functions refers to strengthened phospholipid binding, phosphatidylinositol binding, SH3 domain binding, actin binding, enzyme agonist activation, and Ras GTP enzyme binding, which are related to the activity of macrophages and the activation of related signaling pathways. These results imply that, as a myeloid leukocyte, macrophages have strong cell migration and actin skeleton formation abilities and are able to synthesize multiple vesicles, possibly interacting with the hematopoietic microenvironment of HSCs through endocytosis and exocytosis.

A closer analysis of transcription regulation and cell communication, as shown in Supplementary Figure S3, identified 106 transcription factor genes, 167 surface protein genes, and 116 secretory protein genes as our candidate genes, and the top 20 genes with the highest expression in each category were selected and examined in more depth. Significantly, more than half of the highly expressed genes active in transfection regulations, such as *Junb*, *Fos*, *Id1/2/3*, *C3ar1*, *Mafb, Atf3, Zfp36l1, Cebpb, Klf6, Irf5, Zeb2,* and *Plek,* were involved in hematopoiesis and the homeostasis maintenance of HSCs [26,27,28].

### 3.3. Validation of Esults with Various Methods

**Figure 3:**
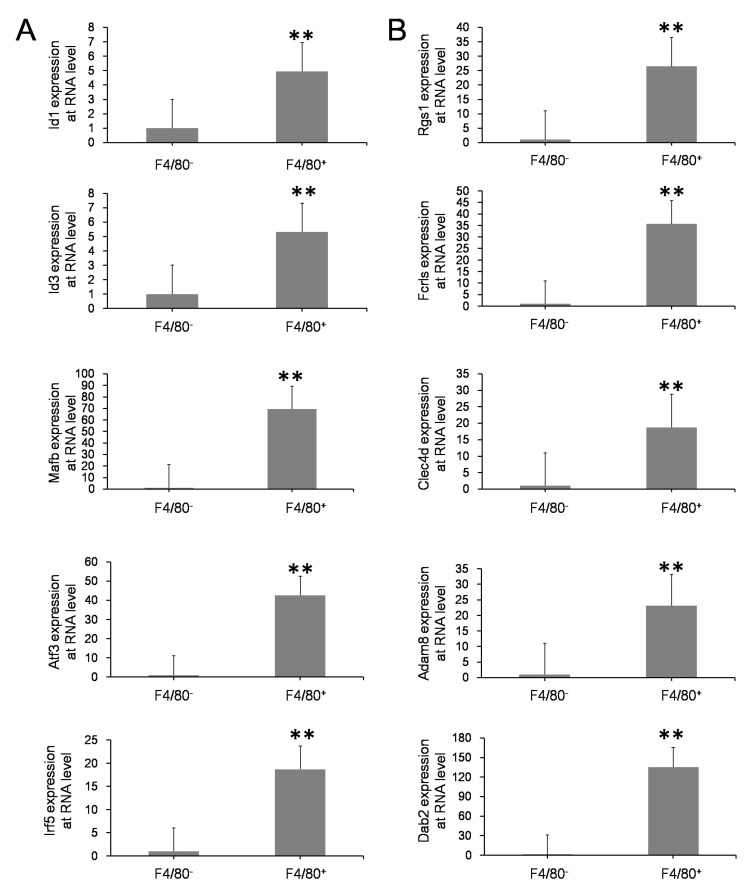
qRT-PCR analysis of key genes with high expression in macrophages. (**A**) The expression of *Id2* and *Id3*, *Mafb*, *Atf3,* and *Irf5* in F4/8^−^ FL-MNCs and F4/80^+^ FL-MNCs. (**B**) The expression of *Rgs1*, *Fcrls*, *Clec4d*, *Adam8,* and *Dab2* in F4/80^−^ FL-MNCs and F4/80^+^ FL-MNCs. ** F4/80^+^ FL-MNCs vs. F4/80^−^ FL-MNCs, *p* < 0.01. Statistical analysis was evaluated with chi-square test.

**Figure 4:**
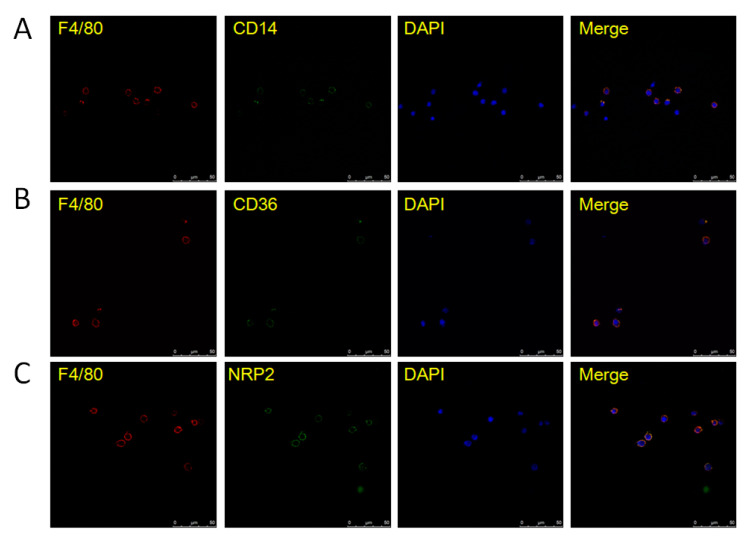
Immunofluorescence results for proteins (CD14, CD36, and NRP2) expressed in F4/80^+^ FL-MNCs. (**A**) CD14 expressed in F4/80^+^ FL-MNCs. (**B**) CD36 expressed in F4/80^+^ FL-MNCs and (**C**) NRP2 expressed in F4/80^+^ FL-MNCs.

The above analysis results were confirmed by different experimental methods at the transcription and translational levels. First of all, qRT-PCR was performed on differentially expressed genes signified in the fetal macrophages. Significant differences were found in the expression of transcription factor (TF) genes, including *Id1*, *Id3*, *Mafb*, *Atf3*, and *Irf5*, and in other genes, including *Adam8*, *Rgs1*, *Fcrls*, *Clec4d*, and *Dab2*, between the F4/80^+^ FL-MNCs and the F4/80^−^ FL-MNCs (Figure 3). This is consistent with the results obtained from the scRNA-seq analysis (Supplementary Figure S3B). The higher expression of the membrane proteins CD14, CD36, NRP2, CSF1R, and MRC; secretion proteins C1QA, C1QB, and C1QC and monocyte–macrophage spectrum marker CD68 showed that macrophages could express a large number of classic genes related to chemokines, complement factors, and various cell receptors that might be important for maintaining a particular microenvironment. Subsequently, confocal images of macrophages indicated that the aforementioned membrane proteins, CD14, CD36, and NRP2, which are typical surface markers of macrophages, were indeed expressed on the surface of most F4/80-positive cells (Figure 4).

**Figure 5:**
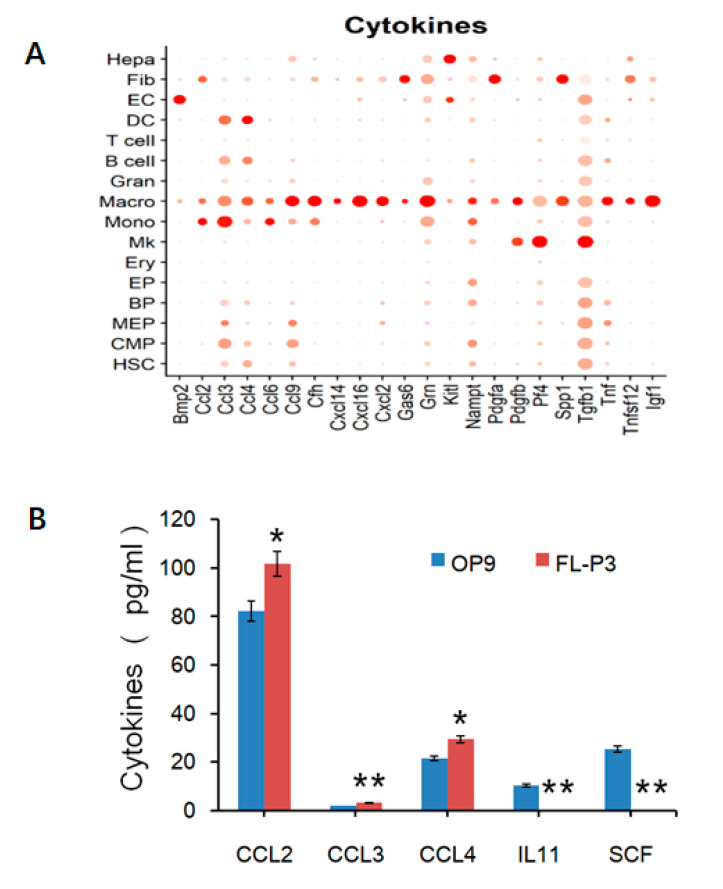
ELISA confirmation analysis of cytokines. (**A**) Bioinformatics analysis of genes with high expression of cytokines in fetal liver macrophages. (**B**) Detection of cytokine expression in the two samples (Blue for OP9 and Red for FL-P3) using ELISA method. FL-P3 vs. OP9: * *p* < 0.05; ** *p* < 0.01. Statistical analysis was evaluated with chi-square test.

As cytokines secreted by macrophages have diverse roles in the maintenance, homing, differentiation, and other aspects of the pluripotency of HSCs [1], an ELISA assays on cytokines were used to validate the results of the scRNA-seq analysis (illustrated in Figure 5).

OP9 cells have been previously reported for their use as feeder cells to maintain their ability to support hematopoiesis and support HSCs differentiation into various hematopoietic cells [29,30,31], though they cannot express or secrete the macrophage-colony-stimulating factor (M-CSF) [32]. Therefore, OP9 cells were be used as reference cells to analyze the cytokines’ characteristics. Macrophages were noted to express more chemokines than other blood cells, such as CCL2, CCL3, CCL4, etc. (Figure 5A). Quantitative analysis of secreted proteins in the supernatant of the FL-P3 cell culture compared to that of OP9 cells revealed higher levels of CCL2, CCL3, CCL4, and the ELISA results also showed lower levels of inflammatory factor IL-11, and almost no expression of SCF (Figure 5B). This indicates that fetal liver macrophages can mediate cell migration but with low immune function. The quantitative detection of these cytokines could help us to better understand these special cells’ fate with respect to their regulation by HSCs and macrophages.

## 4. Discussion

In the course of hematopoietic development, it was found that hematopoietic activity began in the yolk sac and generated hematopoietic progenitor cells in the AGM region, which were later transferred to the fetal liver, where the HSCs expanded massively and settled into the bone marrow. Therefore, different microenvironments could affect the characteristics of HSCs and certain interactions take place between microenvironment cells and HSCs. In this study, a series of approaches were successfully conducted to investigate the possible mechanisms of HSCs and their microenvironment cells with various methods. FL-MNCs, BM-MNCs, and FL-P3 samples were selected, and scRNA-seq technology was used to analyze three samples at the single-cell level. After the integration analysis of the three samples, 17 cell types were obtained, and divided into three categories: 10 types of myeloid cells, 4 types of lymphocytes, and 3 types of other cells.

There was a higher proportion of hematopoietic stem progenitor cells in the FL-MNCs had a higher proportion compared with the BM-MNCs, which highly expressed *Hmga2*, *Npm1*, *Ncl,* and other genes related to the efficient expansion of HSCs. *Mif*, a cytokine that can promote macrophage retention, was found to have a high expression level. It has been reported that *Mif* was highly expressed in many tumor cells and promoted tumor growth [33,34], and its content in serum after HSC transplantation is said to aggravate the graft versus host reaction [35]. Another research team reported that lacking CD74 (a MIF protein receptor) led to exhibited an accumulation of HSCs in the bone marrow due to their increased potential to repopulate and compete in a BM microenvironment [36]. Studies carried out in the last two years have shown that there is indeed spatial contact between macrophages and HSCs, which may be involved in the regulation of the steady state and differentiation of HSCs [37,38].

Macrophages, which are immune cells with phagocytic functions, have tissue specificity and can maintain the settlement of HSCs in the bone marrow, affecting the mobilization of HSCs into peripheral blood after drug stimulation [39]. During embryonic development, CD206^+^ macrophages in the AGM region accumulate around hematogenic endothelial cells, inducing the EHT process [40] and being closely related to the maturation of HSCs [41]. Additionally, mature HSCs are guided by VCAM-1^+^ macrophages and homing to the tail hematopoietic microenvironment for expansion [42]. Our analysis results showed that macrophages can be divided into four groups, accounted for the largest proportion of fetal liver stromal cell populations cultured in vitro and had strong proliferative capacity. However, there were very few macrophages in the fetal liver, and the in vitro culture system can make up for the insufficient quantity and be more effective in single-cell sequencing analysis. Macrophage marker genes analysis inferred that they might be involved in the homeostasis regulation of the hematopoietic microenvironment in HSCs migration or HSCs entering blood vessels, thereby completing extramedullary to intramedullary transition.

The GO analysis of differentially expressed genes between macrophages from FL-MNCs vs. BM-MNCs (Supplementary Figure S3A) and FL-P3 vs. BM-MNCs (Supplementary Figure S3B) showed similar results, indicating that macrophages from FL-MNCs and FL-P3 are somewhat similar in terms of function. Further pathway analysis of differentially expressed genes suggested that the chemokine signaling pathway is more predominant in FL-P3; this might be the cause of HSCs migration. Meanwhile, the mRNA processing pathway is more obvious in FL-MNCs, which might be related to the maintenance of macrophage stability. These two different signaling pathways reflect the need for macrophages to adapt to the environment. Unpolarized macrophages can be polarized and divided into M1 macrophages and M2 macrophages under certain conditions. Supplementary Figure S4 shows that fetal liver macrophages were mostly inactive M0 macrophages, which are unpolarized and highly associated with maintaining homeostasis in the internal environment. M0 macrophages can undergo polarization under stimuli such as inflammation [43]. Studies have shown that M2 macrophages formed after IL-4 activation can effectively promote the proliferation of HSCs [44]. Badham reported that exposure to environmental pollutants in the uterus can increase reactive oxygen species in fetal liver cells, disrupt hematopoietic signaling pathways, and cause childhood leukemia [45]. Macrophages can clear HSCs with highly reactive oxygen species, thereby ensuring the stability and quality of HSCs [38]. Corresponding to the genes in Supplementary Figure S3B, qRT-PCR analysis was performed on the transcription factors, immunofluorescence staining was performed on the surface markers, and ELISA was performed on the secreted factors. These data further confirmed that the expression of genes screened via single-cell RNA sequencing in macrophages was significantly higher than that in non-macrophages. These analysis results suggest that fetal liver macrophages have an important regulatory role on HSCs, which may determine the fate of hematopoietic stem cells.

## 5. Conclusions

In this paper, cellular compositions from different development stages of HSCs originating from the fetal liver, bone marrow, and *in vitro*-cultured fetal liver stromal cells were compared. It was found that the fetal liver hematopoietic microenvironment is more conducive to the proliferation and self-renewal of HSCs. Furthermore, the fetal liver macrophages can be enriched, and a large number of these specific cells can be amplified through culture *in vitro*. The single-cell RNA sequencing analysis presented here with systematical confirmation can be used to reveal the multiple effects of fetal liver macrophages on steady-state maintenance, expansion, and interaction with HSCs. Our work, as exemplified by a study of HSCs during different stages of hematopoietic development, could also be expanded to studies in other areas, such as the physiology or pathology of cancer.

## Data Availability

The data presented in this study have been submitted to the website for China National Center for Bioinformation (https://ngdc.cncb.ac.cn/gsub/) (GSA No. CRA013118).

## Supplementary Materials

The following supporting information can be downloaded at https://www.mdpi.com/article/10.3390/life13112157/s1. Figure S1. Data features of BM-MNC, FL-MNC, and FL-P3 samples before and after cell filtration; Figure S2. Differentially expressed genes of each cell cluster in BM-MNC and FL-MNC samples; Figure S3. Bioinformatics analysis of genes with highly expressed genes in fetal liver macrophages; Figure S4. The cytological characteristics of macrophages in fetal liver; Table S1. Sequences of forward primers and reverse primers used in qRT-PCR; Table S2. Statistics of scRNA-seq results for BM-MNC, FL-MNC, and FL-P3 samples; Table S3. Sequence comparison results of sequencing data quality control for BM-MNC, FL-MNC, and FL-P3 samples; Table S4. Cell and gene quantitative results of sequencing data quality control for BM-MNC, FL-MNC, and FL-P3 samples; Table S5. Annotated results for each cell subgroup and information on marker genes; Table S6. Detailed list of GO enriched genes.
