# Supplementary material for: Identification of Cellular Compositions in Different Microenvironments and Their Potential Impacts on Hematopoietic Stem Cells HSCs Using Single-Cell RNA Sequencing with Systematical Confirmation
Source: Life (Basel). 2023 Nov 2;13(11):2157. doi: 10.3390/life13112157 (PMC10671877; doi:10.3390/life13112157)
Supplement: Supplementary file 1 [file life-13-02157-s001.zip › life-2611578-supplementary.pdf]

Supplementary Materials

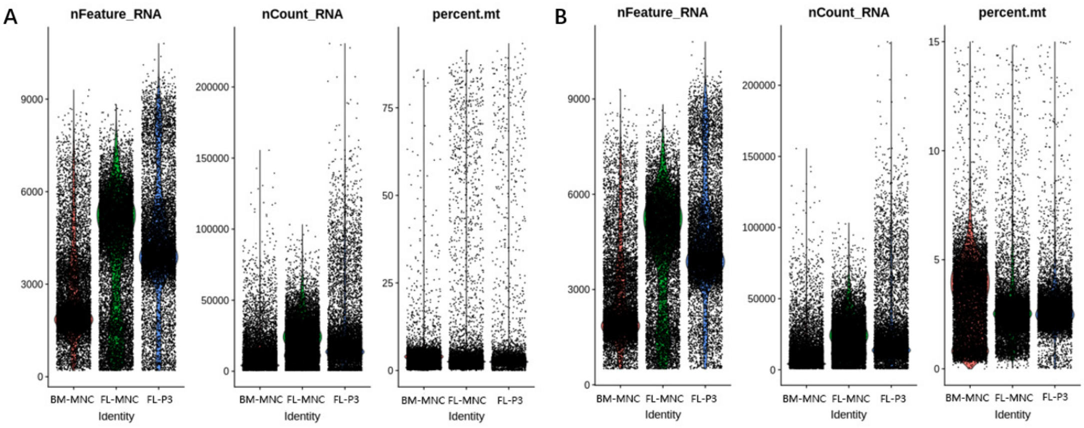

Figure S1. Data features of BM-MNCs, FL-MNCs and FL-P3 samples before and after cell filtration. (**A**) A violin chart showing basic cell information of data before cell filtration, in bone marrow (BM), fetal liver (FL) and FL-P3 (**B**) A violin chart showing basic cell information in BM, FL and P3 sample data after cell filtration. Where nFeature_RNA represents the number of genes detected in a single cell, nCount_RNA represents the number of UMI detected in a single cell, and percent.mt represents the percentage of mitochondrial gene expression in a single cell.

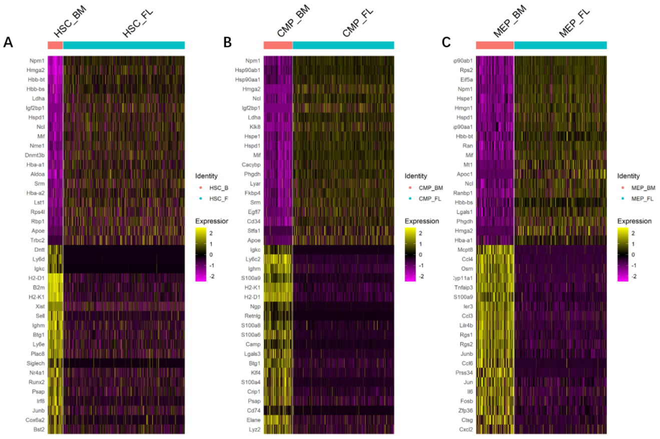

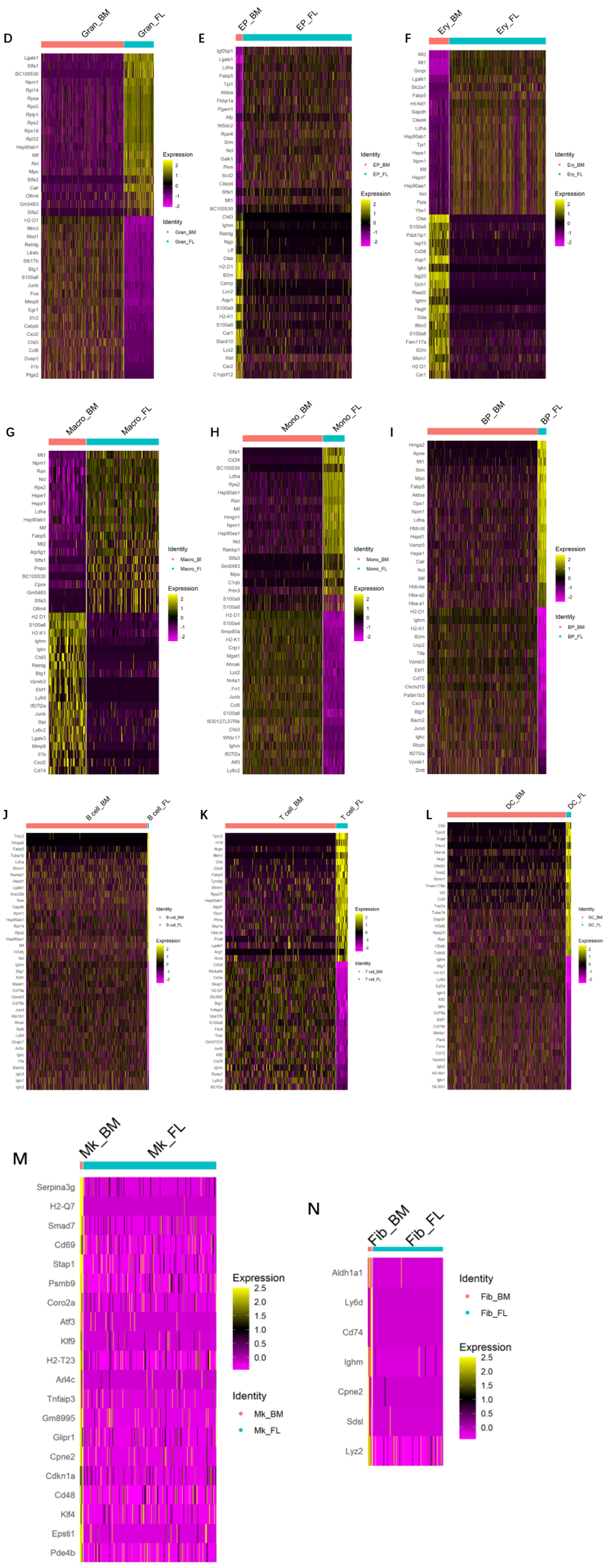

Figure S2. Differentially expressed genes of each cell cluster in BM-MNCs and FL-MNCs samples. (A)-(N) Heat maps showing genes differentially expressed in two samples in a subset of cells common to

the BM-MNCs and FL-MNCs samples, respectively. The (A) subsets of HSCs, (B) subset of CMP, (C) subset of MEP, (D) subset of Gran, (E) subset of EP, (F) subset of ERY, (G) subset of Macro, (H) subset of Mono, (I) subset of BP, (J) subset of B cell, (K) subset of T cell, and (L) subset of DC all showed the 20 genes with the highest differential expression in the BM-MNCs and FL-MNCs samples, respectively. (M) Subgroup Mk, showing only the 20 genes with the highest expression in the BM-MNCs sample because there were no differential genes with significantly high expression in the FL-MNCs sample. (N) Subgroup of Fib, showing only 7 genes overexpressed in the BM-MNCs sample because there were no differential genes significantly overexpressed in the FL-MNCs sample. Wherein each row represents a gene, and each column rep-resents a cell; a yellow color indicates higher gene expression; and a red color indicates lower gene expression.

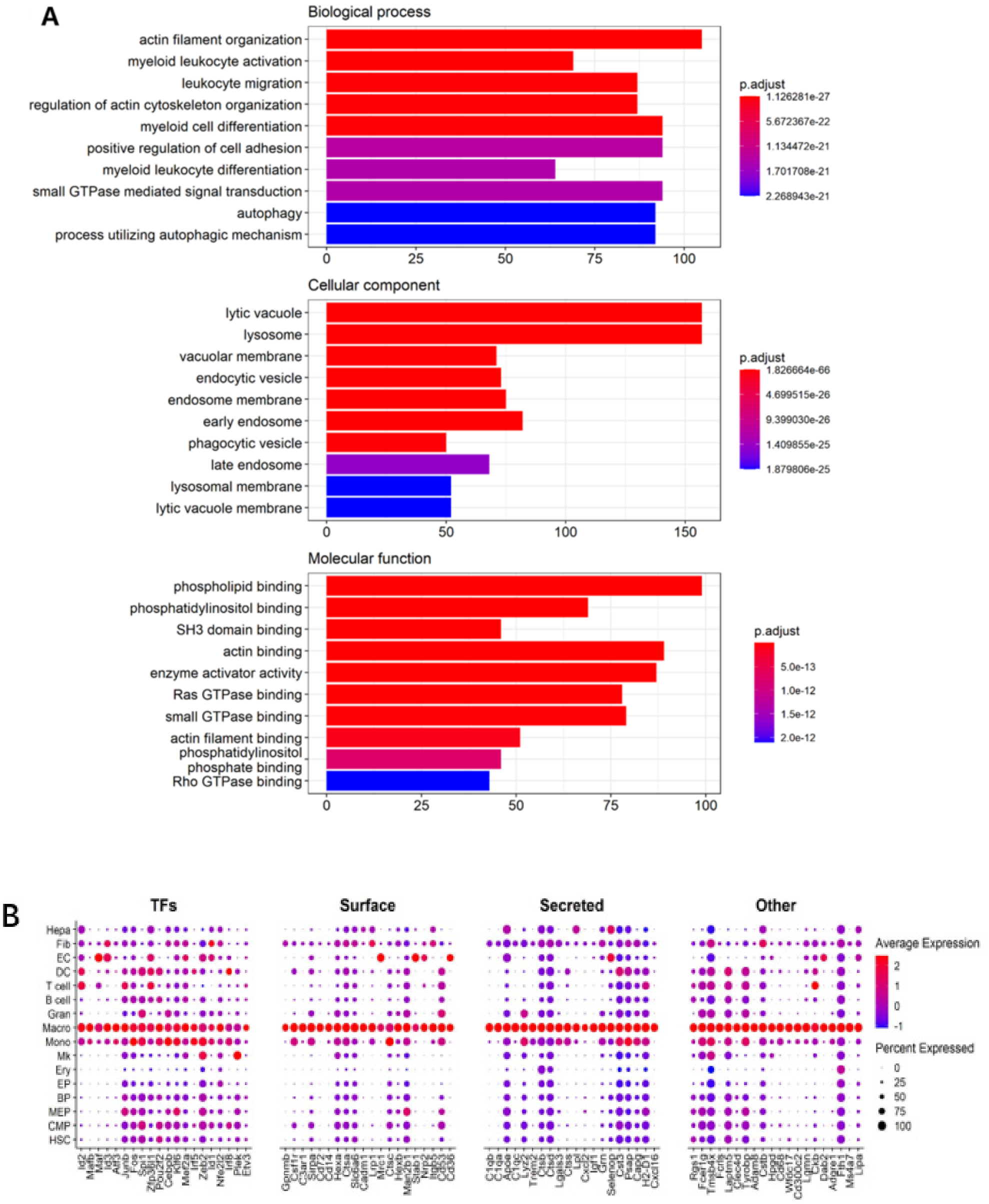

Figure S3. Bioinformatics analysis of genes with high expression genes in fetal liver macrophages. (**A**) GO analysis of function enrichment of up-regulated genes in fetal liver macrophages. The bar chart shows the GO items enriched by genes with high expression of macrophages, which are enriched in biological process, cell composition and molecular function from top to bottom, with each line

representing a GO item; The abscissa represents the number of genes enriched in this item; The red color indicates that the P value is smaller and more significant. (**B**) Bioinformatics analysis of genes with high expression of transcription factors, surface proteins and secreted proteins in fetal liver macrophages. The bubble chart shows the expression of high-expression genes in transcription factors and cell communication of macrophages. TFs refers to transcription factor; Surface refers to surface proteins; Secreted refers to secretion proteins; and Other represents the rest of the high-expression genes, each of which displays the 20 genes with the highest expression. Red color indicates the higher expression level of this gene, and a larger dot indicates the higher expression ratio of this gene in this cell subpopulation.

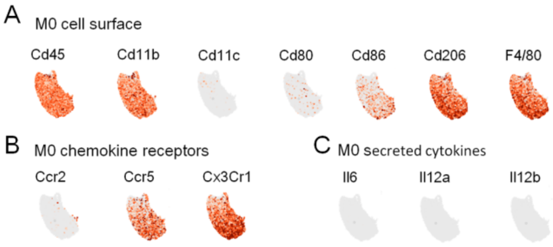

Figure S4. Identify the cytological characteristics of macrophages in fetal liver. A. M0 cell surface gene expression character: effective expression genes included Cd45, Cd11b, Cd86, Cd206 and F4/80, genes that are not expressed or are very weakly expressed included Cd11c and Cd80. B. M0 chemokine receptor gene expression character: effective expression genes included Ccr5 and Cx3cr1, genes that are very weakly expressed included Ccr2. C. M0 secreted cytokine gene expression character: genes that are not expressed included Il6 and Il12 (Il12a and Il12b).

Table S1. Sequences of forward primers and reverse primers used in qRT-PCR

Gene	Direction	Nucleotide Sequence (5′ to 3′)
β-actin	Forward	CATCCGTAAAGACCTCTATGCCAAC
	Reverse	ATGGAGCCACCGATCCACA
Id1	Forward	CCTAGCTGTTCGCTGAAGGC
	Reverse	CTCCGACAGACCAAGTACCAC
Id3	Forward	CTGTCGGAACGTAGCCTGG
	Reverse	GTGGTTCATGTCGTCCAAGAG
Mafb	Forward	TTCGACCTTCTCAAGTTCGACG
	Reverse	TCGAGATGGGTCTTCGGTTCA
Atf3	Forward	GAGGATTTTGCTAACCTGACACC
	Reverse	TTGACGGTAACTGACTCCAGC
Irf5	Forward	GGTCAACGGGGAAAAGAAACT

	Reverse	CATCCACCCCTTCAGTGTACT
Rgs1	Forward	TCTGGGATGAAATCGGCCAAG
	Reverse	GCATCTGAATGCACAAATGCTT
Fcrls	Forward	CTTCTGGTCTTCGCTCCTGTC
	Reverse	ATGGTGTAGCTTGAAGCACTG
Clec4d	Forward	ACCCGACATCCCCAACTGAT
	Reverse	CTCTCGTCCAGCGTAAAAAGT
Adam8	Forward	TTGCCCCATGTGAAACAGTATG
	Reverse	AGGTGCAGGGTGAAAACGTG
Dab2	Forward	CCTTCATTGCTCGTGATGTGA
	Reverse	CCCCAAACAAATCCATCTGGTC

Table S2. Statistics of scRNA-seq sequencing results for BM-MNCs, FL-MNCs, and FL-P3 samples

	BM-MNCs	FL-MNCs	FL-P3
Number of Reads	815,828,847	823,536,495	781,776,201
Valid Barcodes	96.0 %	97.5 %	97.3 %
Sequencing Saturation	66.2 %	36.4 %	39.0 %
Q30 Bases in Barcode	96.3 %	96.5 %	96.0 %
Q30 Bases in RNA Read	93.3 %	94.0 %	93.5 %
Q30 Bases in UMI	94.7 %	95.0 %	93.5 %

Number of Reads: The number of reads obtained from sample sequencing; Valid Barcodes: Ratio of the data with correct 10x Barcodes, each Barcode corresponds to each cell; Q30 Bases in Barcodes: The percentage of bases with a mass fraction in the Barcode sequence; Q30 Bases in RNA Read: The percentage of bases with a mass fraction equal to or greater than 30% in the Reads used for comparison; Q30 Bases in UMI: The percentage of bases in the UMI sequence with a mass fraction equal to or greater than 30%.

Table S3. Sequence comparison results of quality controls of sequencing data for BM-MNCs, FL-MNCs, and FL-P3 samples

	BM-MNCs	FL-MNCs	FL-P3
Reads Mapped to Genome	91.6 %	94.0 %	95.1 %
Reads Mapped Confidently to Genome	80.9 %	79.8 %	84.3 %
Reads Mapped Confidently to Intergenic Regions	2.8 %	2.4 %	2.0 %
Reads Mapped Confidently to Intronic Regions	22.7 %	13.9 %	14.5 %
Reads Mapped Confidently to Exonic Regions	55.5 %	63.5 %	67.8 %
Reads Mapped Confidently to Transcriptome	51.9 %	60.3 %	64.2 %
Reads Mapped Antisense to Gene	1.8 %	1.3 %	1.5 %

Reads Mapped to Genome: The ratio of reads mapped to reference genome; Reads Mapped Confidently to Genome: The proportion of reads that are matched to the reference genome and supported by GTF information; Reads Mapped Confidence to Integer Regions: The ratio of reads mapped to inter gene regions; Reads Mapped Confidence to Intronic Regions: The ratio of reads mapped to intronic regions; Reads Mapped Confidence to Exonic Regions: The ratio of reads mapped to exon regions; Reads Mapped Confidently to Transcriptome: The ratio of reads mapped to a known reference transcript; Reads Mapped Anti sense to Gene: The ratio of reads mapped to the gene antisense chain.

Table S4. Cell and gene quantitative results of quality controls of sequencing data for BM-MNCs, FL-MNCs, and FL-P3 samples

	BM-MNCs	FL-MNCs	FL-P3
Estimated Number of Cells	11,627	13,373	11,015
Fraction Reads in Cells	95.20 %	95.50 %	94.60 %
Mean Reads per Cell	70,166	61,582	70,973
Median Genes per Cell	2,104	4,619	4,133
Total Genes Detected	28,154	29,974	30,575
Median UMI Counts per Cell	5,714	21,190	16,108

Estimated Number of Cells: The effective number of cells detected in each sample; Fraction Reads in Cells: The proportion of reads that are compared to the reference gene and originate from high-quality cells; Mean Reads Per Cell: Average reads per cell; Median Genes Per Cell: The median number of genes detected in each cell; Total Genes Detected: The total number of genes detected in all cells; Median UMI Counts per Cell: The median number of UMI detected by each cell.

Table S5. Information on annotated results of each cell subgroup and marker genes

Cell Type No.	Abbreviation of Cell Type	Full Name of Cell Type	Marker Gene
10	HSCs	Hematopoietic Stem cells	*Cd34, Kit, Ly6a, Hlf*
2, 38	CMP	Common myeloid progenitor	*Cd34, Kit, Plac8*
27, 39	MEP	Megakaryocyte and Erythrocyte Progenitors	*Cd34, Kit, Gata2, Tfrc*
5, 12, 26, 28	EP	Erythroid progenitors	*Kit, Tfrc, Cited4, Car2*
4, 11, 15, 23	Ery	Erythroid	*Hba-a1, Hba-a2, Hbb-bs, Hbb-bt*
31	Mk	Megakaryocyte	*Pf4, Itga2b, Itgb3*
16	Mono	Monocyte	*F13a1, Ly6c2*
3, 6, 7, 8, 13, 24, 37	Macro	Macrophage	*Emr1, C1qb, Ms4a7*
18, 19, 20, 33, 36	Gran	Granulocyte	*Ly6g, S100a8*
17, 29	BP	B cell progenitors	*Igll1, Vpreb2*
0, 9, 21	B cell	B cell	*Vpreb3, Ebf1, Cd79a*
30	T cell	T cell	*Trbc2, Cd3e,*
34	NK	Natural killer cell	*Gzma, Ncr1*
14, 32	DC	Dendritic cell	*Cd74, H2-Aa*
35	EC	Endothelial cell	*Cdh5, Kdr, Lyve1*
1, 22	Fib	Fibroblast	*Col1a1, Acta2, Fn1*
25	Hepa	Hepatoblast	*Afp, Alb, Dlk1*
